# Use of Questionnaire-Based Measures in the Assessment of Listening Difficulties in School-Aged Children

**DOI:** 10.1097/AUD.0000000000000180

**Published:** 2015-10-27

**Authors:** Johanna G. Barry, Danielle Tomlin, David R. Moore, Harvey Dillon

**Affiliations:** 1 MRC Institute of Hearing Research, University Park, Nottingham, United Kingdom; 2 Department of Audiology and Speech Pathology, The University of Melbourne, Melbourne, Australia; 3 The HEARing Cooperative Research Centre, Victoria, Australia; 4 Communication Sciences Research Center, Cincinnati Children’s Hospital and Department of Otolaryngology, University of Cincinnati, Cincinnati, Ohio, USA; and 5 National Acoustic Laboratories, Sydney, Australia.

**Keywords:** Assessment, Auditory processing, Auditory processing disorder, Clinical test battery, Cognition, Questionnaires

## Abstract

In this study, the authors assessed the potential utility of a recently developed questionnaire (Evaluation of Children’s Listening and Processing Skills [ECLiPS]) for supporting the clinical assessment of children referred for auditory processing disorder (APD).

**Design:**

A total of 49 children (35 referred for APD assessment and 14 from mainstream schools) were assessed for auditory processing (AP) abilities, cognitive abilities, and symptoms of listening difficulty. Four questionnaires were used to capture the symptoms of listening difficulty from the perspective of parents (ECLiPS and Fisher’s auditory problem checklist), teachers (Teacher’s Evaluation of Auditory Performance), and children, that is, self-report (Listening Inventory for Education). Correlation analyses tested for convergence between the questionnaires and both cognitive and AP measures. Discriminant analyses were performed to determine the best combination of tests for discriminating between typically developing children and children referred for APD.

**Results:**

All questionnaires were sensitive to the presence of difficulty, that is, children referred for assessment had significantly more symptoms of listening difficulty than typically developing children. There was, however, no evidence of more listening difficulty in children meeting the diagnostic criteria for APD. Some AP tests were significantly correlated with ECLiPS factors measuring related abilities providing evidence for construct validity. All questionnaires correlated to a greater or lesser extent with the cognitive measures in the study. Discriminant analysis suggested that the best discrimination between groups was achieved using a combination of ECLiPS factors, together with nonverbal Intelligence Quotient (cognitive) and AP measures (i.e., dichotic digits test and frequency pattern test).

**Conclusions:**

The ECLiPS was particularly sensitive to cognitive difficulties, an important aspect of many children referred for APD, as well as correlating with some AP measures. It can potentially support the preliminary assessment of children referred for APD.

## Abstract 2

Questionnaires potentially offer valuable information about everyday listening difficulty in children referred for suspected APD. However, there are many problems with currently available questionnaires. This paper considers the validity and usefulness of a new questionnaire called the Evaluation of Children’s Listening and Processing Skills (ECLiPS) in comparison with three other available questionnaires. All the questionnaires in the study were similarly sensitive to the presence of problems, but the ECLiPS provided clearer evidence of construct validity particularly with respect to measures of cognitive abilities. Discriminant analysis suggested that a combination of ECLiPS factors with auditory and cognitive measures achieved best discrimination among the participant groups in the study.

## INTRODUCTION

Some children with normal audiometric thresholds and no known etiology, neurological pathology, or other underlying risk factor have disproportionate difficulty processing speech, particularly in noisy conditions. Because of the apparent auditory nature of their difficulties, these children are often referred to a pediatric audiologist for assessment for developmental auditory processing disorder (APD). This disorder is distinct from other APDs, which can be attributed to some external factors such as neurological trauma (acquired APD) or hearing impairment (secondary APD).

While there is certainly an auditory component to the difficulties that these children experience (e.g., Moore 2006), it is not clear that their difficulties are specifically auditory in nature because the children often also display problems with short-term memory and attention, as well as having poorer language, literacy, and social skills. As a consequence, these children represent a significant challenge to clinical practice. For one thing, it is not clear who is the most appropriate health professional that they should be referred to. The trend, at present, is to recommend assessment by a multidisciplinary team (e.g., American Speech-Language Hearing Association [ASHA] 2005; Dawes & Bishop 2009). For another thing, it is not clear what measures should be used in the assessment of the child or what cutoff criteria to apply for establishing a diagnosis of APD (i.e., a clinically significant listening difficulty) (Hind 2006; Dillon et al. 2012; Wilson & Arnott 2013). Design of clinical test batteries largely reflects different theoretical conceptions about the nature and cause of the listening difficulties. Choice of cutoff criteria for determining diagnosis depends in the clinical context on current recommendations from professional bodies (e.g., ASHA 2005; American Academy of Audiology [AAA] 2010), while in the research context, it will reflect the theoretical position of the researcher and consequently varies across studies (Wilson et al. 2011).

Reflecting the variability in clinical test batteries and applied cutoff criteria, as well as the lack of any accepted gold standard test specifically sensitive to APD, confidence in either the reliability or validity of a diagnosis of APD is low among clinical and research professionals. As a consequence, at least in the United Kingdom, few pediatric audiology services are willing to assess children referred for suspected APD (Hind 2006). This is unsatisfactory for all concerned, but particularly for the affected children and their families, because there is no doubt that these children do have difficulties that, in some cases, can be quite debilitating.

The question therefore arises: how should children with listening difficulties be assessed?

Ideally, a well-structured clinical assessment will carefully probe for relevant symptoms as a first step toward establishing a diagnosis and developing an appropriate treatment plan. In the case of APD, however, it is becoming increasingly clear that the associated listening difficulties are not unique to the disorder. Moreover, despite numerous attempts to demonstrate otherwise (Dawes et al. 2008; Sharma et al. 2009; Ferguson et al. 2011; Miller & Wagstaff 2011), available evidence suggests that the difficulties observed in children receiving a diagnosis of APD are not readily distinguishable from those observed in children with other diagnoses, such as specific language impairment (e.g., Ferguson et al. 2011), specific reading impairment (Dawes & Bishop 2009), or attention deficit disorder (Ghanizadeh 2009). Consequently, definitions of developmental APD have shifted from delineating what makes the disorder unique (e.g., British Society of Audiology 2007) to acknowledging that it can occur in the context of other developmental disorders (e.g., British Society of Audiology 2011). There is now a discussion about how symptoms of listening difficulties relate to other developmental disorders (Miller & Wagstaff 2011). For example, Dillon et al. (2012) accept that there are shared symptoms among disorders and argue that clinicians should focus on identifying the specific deficits underlying an individual child’s listening difficulties. Moore and Hunter (2013), however, suggest that all symptoms of difficulty with listening, language, and literacy may represent different manifestations of a single underlying neurodevelopmental syndrome. From a practical stand point, these debates undermine the possibility of achieving one of the key goals for a clinical assessment, namely, the diagnosis of the disorder. Dillon et al. (2012) have consequently argued that rather than aiming for diagnosis, clinicians should focus instead on applying a systematic, hierarchically structured assessment procedure to carefully document the range and severity of difficulties experienced by an individual, as well as, where possible, the specific underlying deficit. As such, this approach explicitly refocuses attention on the individual and on developing an appropriately targeted remediation strategy for that individual.

Accepting that testing should be systematic and hierarchically organized implies that the knowledge base exists for making informed decisions regarding the choice of measures, whether behavioral or report based, to include at each level in such a test battery. It also presupposes that the measures included will have well-understood psychometric properties, that is, that they are (a) valid (measure latent traits specifically associated with (un)successful real-world listening) and (b) reliable (consistently sensitive to the trait that they are designed to measure, both within and among individuals).

In practical terms, and despite more than 40 years of research, the body of knowledge for establishing the best set of tests for assessing the real-world listening difficulties still does not exist (Dillon et al. 2012; Protopapas 2014). Many clinically available auditory processing (AP) tests are “sensitized” (Cacace & McFarland 2005) by manipulating the speech quality (Hodgson 1972) or the task demands (Berlin & Lowe 1972) to stress language or memory systems. Such strategies effectively undermine test validity by complicating interpretation regarding the latent trait being probed. Once stresses to supramodal abilities are removed, as Moore et al. (2010) have shown, most AP tests become relatively insensitive to the problems that have led to the child being referred.

There are also considerable doubts about the reliability of many tests of auditory abilities because test performance can be highly influenced by age (Moore et al. 2011; Tomlin et al. 2014), auditory experience (Barry et al. 2013), or the cognitive skills brought to bear on the task (e.g., Barry et al. 2012). Moreover, in the clinical context, AP tests have proven singularly uninformative about the real-world listening difficulties leading to referral to a pediatric audiologist. It has consequently been argued that a well-designed and validated questionnaire may be better suited to capturing the everyday listening difficulties that define children referred for assessment for APD (Ferguson et al. 2011; Moore 2012).

Report-based measures (scales/checklists/questionnaires) are relatively inexpensive and easy to administer, as well as being potentially powerful tools for supporting clinical assessment (Wilson et al. 2011). Because the same measures can be administered to multiple respondents, clinicians can also use them to develop a broader insight into the range and severity of a child’s real-world listening problems in different contexts. All of this presupposes, of course, that the measure has the requisite properties of psychometric reliability and validity. Psychometric validity requires the inclusion of a range of items that are sufficiently informative of different aspects of the latent trait of interest. Psychometric reliability requires that each item consistently measures the same trait both within respondents at different time points (test–retest) and across respondents (inter-rater). This is achieved, first, by including items that all respondents are sensitive to, and second, by ensuring that all items are readily understandable and minimally ambiguous regarding interpretation.

Despite their potential strengths, however, report-based measures are liable to problems of subjectivity and response bias (Wilson et al. 2011). These problems cannot be excluded but can be minimized by careful design.

A number of questionnaires currently exist for assessing children with listening difficulties (e.g., Fisher 1976; Anderson 1989; Smoski et al. 1992; Meister et al. 2004). With the exception of some recent research (Iliadou & Bamiou 2012), reliable relationships have yet to be demonstrated between these questionnaires and clinical AP tests, even though the two types of measure are assessing apparently related difficulties (Lam & Sanchez 2007; Moore et al. 2010; Wilson et al. 2011). More seriously, with the possible exception of the questionnaire developed by Meister et al. (2004), none of the currently available questionnaires has been systematically designed in accordance with the principles of good scale development. Typically, the questionnaires have emerged as a means for summarizing symptoms regularly observed by clinicians. Response scales have then been appended to gauge severity. As a consequence, questionnaires do not approach the level of psychometric robustness required for reliably assessing either the severity or the nature of a child’s listening difficulties.

To address the lack of a well-designed and validated measure of symptoms associated with APD, Barry and Moore (2014) developed the Evaluation of Children’s Listening and Processing Skills (ECLiPS) by applying a carefully specified and systematic procedure for development (Cronbach & Meehl 1955). This approach required the following: (1) specification of the psychological construct (latent trait(s) to be measured), (2) development of a sufficiently broad-ranging but carefully worded item pool, and (3) assessment of the construct validity and reliability of measurement of the final scale. The British Society of Audiology (2011) position statement for APD provided the basis for specifying the psychological construct for the ECLiPS. This statement explicitly accepts that affected children have difficulties in processing both nonspeech and speech stimuli. It also recognizes the contribution of higher cognitive abilities to listening difficulties and acknowledges the common co-occurrence of developmental APD with other language-based learning problems. The aim of this study was to extend on this initial development of the ECLiPS to consider what role, if any, the questionnaire could play in the clinical assessment of children referred for suspected APD.

If an assessment tool is to be clinically useful, in addition to being a sensitive and valid assessment of difficulty, it must also be efficient in terms of cost/time to administer, relative to information obtained through using it. With these considerations in mind, our study had three main subquestions:

- To what extent do scores on the ECLiPS converge with other currently used questionnaires?
- Does the ECLiPS demonstrate a greater measurement validity compared with other questionnaires (specifically convergent validity) with respect to commonly used clinical AP tests?
- Do the different ECLiPS factors demonstrate convergence/divergence with cognitive measures predicted to tap into the same/different latent traits?

## MATERIALS AND METHODS

This study formed part of a larger study undertaken by the second author (Tomlin 2014), which was designed to assess listening and cognitive abilities in children referred for assessment by pediatric audiologists. It was approved by the Royal Victorian Eye and Ear Hospital Human Research Ethics Committee.

### Participants

The study comprised 49 children ranging in age from 7.1 to 12.8 years who were recruited from the Melbourne metropolitan area. The clinical group comprised 35 children (11 female) who were recruited after being referred to the University of Melbourne Audiology Clinic for further assessment because of suspected APD. Based on performance on a commonly used clinical test battery (described below), 12 of these children (5 female) were identified as meeting diagnostic criteria for APD (ASHA 2005), that is, *z* scores ≤−2 for two or more tasks or ≤−3 on one task (only one child met this latter criterion). These children are referred to as the AP+ group. The remaining 23 children in the clinical group, who did not meet criteria for diagnosis of APD, are referred to as the AP− group.

Performance of the clinical group on all test measures was compared with a group of 14 typically developing (TD) children (10 female) who were recruited from local schools.

All parents completed a case history questionnaire developed in-house, which asked a series of open questions regarding health, history of middle ear disease (otitis media with effusion), concerns about listening ability, reading development, attention, memory, or more general academic progress. The case history questionnaire was used to ensure that (1) the TD children met criteria for normal development and (2) the listening difficulties, for which the clinical groups were referred, did not have an obvious explanation such as a confirmed diagnosis of another developmental disorder. Responses on the questionnaire were operationalized as presence (1) or absence (0) of problem and counts for both groups are summarized in Table 1.

**TABLE 1.**
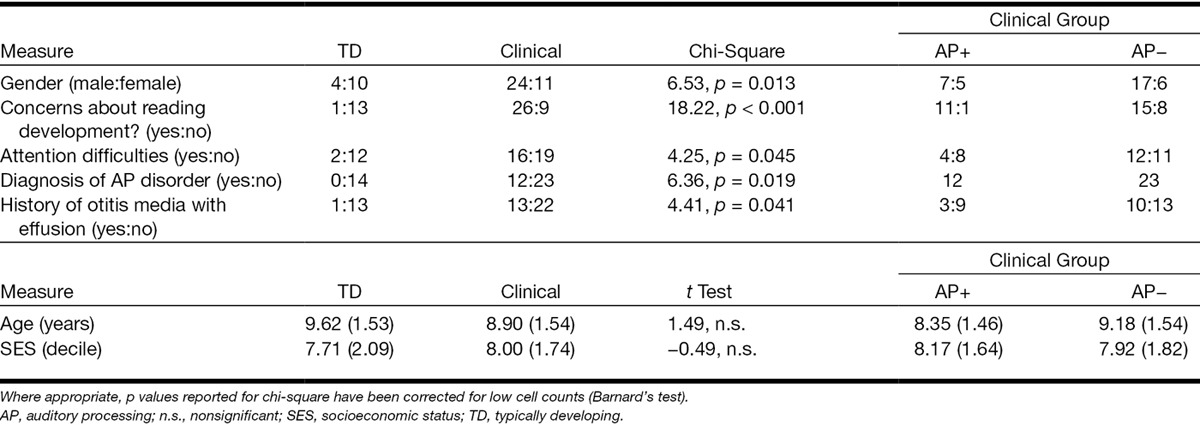
Demographic data for the two groups of children participating in the study (TD and clinical) based on parental information provided in the in-house designed case history questionnaire

All children had normal (≤15 dB HL) pure-tone hearing thresholds (0.5–4 kHz) as assessed using standard audiometric protocols using an A177 Plus portable two channel audiometer (Amplaid) and either TDH 49 headphones or EARtone ER-3A inserts. They also had normal middle ear function and immittance (type A tympanograms defined as a peak compliance within 0.2–1.6 mmho, peak pressure within −100 to +20 dPa, and ipsilateral acoustic reflexes at 1 kHz below 105 dB HL; Jerger 1970). The groups (Table 1) had a comparable mean age and socioeconomic status (Index of Relative Socio-economic Advantage and Disadvantage Australian Bureau of Statistics 2011) but differed significantly on all other measures. Specifically, the clinical group had a higher incidence of both a history of otitis media with effusion and reported difficulties with attention.

### Questionnaires-Based Assessment of Listening Difficulty

Responses for four questionnaires were collected to provide multiple perspectives on the children’s listening abilities. The questionnaires included a self-report measure (the Listening Inventory for Education [LIFE]), a teacher-report measure (the Teacher’s Evaluation of Auditory Performance [TEAP]), and two parental report measures: (a) the ECLiPS and (b) the Fisher’s auditory problem checklist (FAPC). The FAPC, TEAP, and LIFE are commonly used by clinicians in the Australian context to support assessment for listening difficulties.

#### The LIFE: Child Self-Report of Listening Ability

A shortened version (Purdy et al. 2009) of the LIFE (Anderson et al. 2011) was designed to assess listening difficulty in children with hearing losses. In this questionnaire, the child is presented with seven classroom scenarios, for example, “The teacher is talking, but there are children making a noise outside your classroom. How well can you hear the teacher’s words?,” and is asked to indicate on a 5-point Likert-type scale ranging from “Always Difficult” to “Always Easy,” the extent to which listening is difficult for them. Responses are scored from 1 to 5 with higher scores corresponding to greater difficulty. The higher the overall score on this scale, the greater the level of listening difficulty. Data are missing for three TD children.

#### The TEAP

The TEAP (Purdy et al. 2002; Sharma & Purdy 2012) was specifically designed to assess hearing and communication in children using hearing aids and/or a cochlear implant. The teacher is asked to rate the child’s listening ability compared with other children in seven classroom listening environments, for example, “If listening in a quiet room, this child has difficulty hearing and understanding.” Scores range from −5 (cannot function at all) to +1 (less difficulty). The more negative the overall score, the more extensive the range and the level of listening difficulty. TEAP results are not available for 12 children (seven AP− and five TD).

#### The ECLiPS: Parental-Report Measure

The ECLiPS was primarily designed to assess the children with listening difficulties (Barry & Moore 2014). It comprises 37 items, for example, “zones out,” which, based on factor analysis, form five subfactors: (1) Speech and Auditory Processing (SAP), (2) Environmental & Auditory Sensitivity (EAS), (3) Language/Literacy/Laterality (L/L/L), (4) Memory & Attention (M&A), and (5) Pragmatic & Social Skills (PSS). Parents indicate on a 5-point Likert scale, the extent to which they agree or disagree with each statement. Scores for items within each factor are averaged, and age- and gender-scaled scores are calculated.

#### The FAPC: Parental-Report Measure

The FAPC (Fisher 1976) comprises a list of 25 possible difficulties or behaviors, for example, “Is easily distracted by background noise.” Parents indicate which of these they observe in their child. The percentage of behaviors *not observed* is then calculated, with higher scores reflecting less difficulty. Data are missing for three TD children.

#### Questionnaire Validity

To our knowledge, there is relatively little information about the validity of the FAPC, TEAP, and LIFE. However, as part of the larger PhD study (Tomlin 2014), item-total correlations were performed, and items with weak item-total correlations (*r* < 0.3) were removed. Six items were removed from the FAPC (these items were about history of hearing loss or evidence of language delays). One item was removed from the LIFE. It asked about listening during a classroom test, which was a very different listening scenario to the other items in the test. No items were removed from the TEAP. Final Cronbach’s α were FAPC = 0.88, LIFE = 0.74, and TEAP = 0.95.

The ECLiPS has been submitted to an extensive process of validation as part of its development (Barry & Moore 2014). Cronbach’s α for the five ECLiPS factors ranges from 0.83 (PSS) to 0.94 (SAP). The factors have high test–retest reliability (intraclass correlations range from 0.90 [SAP] to 0.96 [PSS] and interrater reliability [parent–parent intraclass correlations] ranges from 0.78 [SAP] to 0.88 [L/L/L]). Finally, assessment of construct validity (Barry & Moore 2014) suggests that the relevant ECLiPS factors have good convergent validity (*r* > 0.5) with listening (Children’s Auditory Performance Scale: Smoski et al. 1992) and language (Children’s Communication Checklist-2: Bishop 2003), as well as reasonable discriminant validity (*r* < 0.35) with the autism-focused Social Communication Questionnaire (Rutter et al. 2003).

#### Standardization for Age

Data obtained on a larger group of similar children from Melbourne indicated no significant effects for age on the FAPC, TEAP, or LIFE, and the raw scores for each were therefore entered into the analyses with the United Kingdom age-normed scores from the ECLiPS.

### Assessment of AP Abilities

#### Protocol for Assessment of AP

The clinical AP test battery comprised five tests commonly used to diagnose APD. Three tests (dichotic digits test [DDT], masking level difference [MLD], and listening in spatialized noise [LiSN-S]) involved binaural presentation of stimuli, while the remaining two (frequency pattern test [FPT] and gaps-in-noise [GIN]) involved monaural presentation of stimuli to each ear separately. Order of presentation of test, and of ear, was randomized among children.

#### Dichotic Digits Test

The DDT (Musiek 1983) involves listening to two pairs of single syllable digits (“seven” excluded) presented to each ear simultaneously at 50 dB HL. Participants report the digits heard at either the right or left ear and the number of correctly identified digits for each ear is scored. There are 40 test items per ear (20 digit pairs).

#### Frequency Pattern Test

The FPT is a measure of temporal sequencing ability (Musiek 1994; Noffsinger et al. 1994). It involves verbally reporting the order of occurrence of a combination of three pure tones (30 triplets at each ear, 150-msec duration, 10-msec rise–fall time, 200-msec intertone interval, 7-sec between different patterns, 50 dB HL). The tones can be either high (H: 1122 Hz) or low (L: 880 Hz) in pitch, and six combinations of tones are possible (HHL, HLH, LHH, LLH, LHL, and HLL). Each combination report is scored as correct or incorrect, with a percent correct score being calculated for each ear.

#### Masking Level Difference

Perception of MLD reflects binaural integration skills, namely, the detection of an interaural phase difference of a low-frequency tone. Pure-tone stimuli (500 Hz) are presented binaurally (50 dB HL) in 3-sec bursts of noise (200–800 Hz) at various signal-to-noise ratios (Wilson et al. 2003). The participant reports whether or not a tone is present in 30 trials. Tones are presented interaurally: (1) in phase (SoNo: homophasic), (2) 180° out of phase (SπNo: antiphasic), or (3) no signal. Release from masking is defined as threshold difference (dB) for the SoNo condition minus the SπNo condition.

#### GIN Test

The GIN test assesses the temporal resolution (Musiek et al. 2005). It comprises a series of 6-sec segments of broadband white noise containing 0 to 3 gaps per noise segment with a minimum intergap interval of 500 msec. Participants press a response button when they hear a “gap” in the noise. The gaps are between 2 and 20 msec in length. Threshold of gap detection (msec) for each ear is determined.

#### LiSN-S Test

The LiSN-S test assesses the spatial release from masking (Cameron & Dillon 2008). Target sentences are presented together with a background noise distractor (a looped discourse [55 dB SPL] of children’s stories), and the participant is required to repeat as many words as possible of each target sentence. The distractors vary in virtual location relative to the target and can be in the same location (at 0°) or orthogonal to it (±90° separation). The test comprises 120 total target sentences with up to 30 target sentences being presented in each of four possible Voice × Location conditions: Same Voice × Same (0°) Location (SV0), Same Voice × Different Location (SV90), Different Voice × Same Location (DV0), and Different Voice × Different Location (DV90). The different conditions are presented in order from least to most difficult, that is, DV90 → SV90 → DV0 → SV0. The signal-to-noise ratio is varied adaptively to determine the participant’s speech reception threshold (dB) for each condition. Five measures are then determined: the LiSN high cue threshold (DV90), the LiSN low cue threshold (SV0), the LiSN spatial advantage (SV0 − SV90), the LiSN tonal advantage (SV0 − DV0), and the LiSN total advantage (SV0 − DV90). Importantly, the spatial, tonal, and total advantage scores are derived scores, so that cognitive contributions common to performance on both measures have no effect on the final score.

#### Standardization and Averaging of Data for Analyses

All AP data were converted to *z* scores to facilitate direct comparison among tests and to identify abnormal performance (*z* score ≤−2) from the normal range of variation.

FPT and DDT scores for each ear were converted to *z* scores (a transformation based on age-specific normative data [Tomlin et al. 2014] where difference from mean of age peers is divided by the standard deviation of that population). Similarly, *z* scores for the GIN and MLD data were generated using the data from 50 control children (Tomlin 2014, PhD Data, Personal communication).

To reduce the amount of data to be included in the analyses, mean *z* scores for DDT, FPT, and GIN were calculated by averaging the *z* scores obtained for each ear.

LiSN-S results were automatically converted to *z* scores by the reporting software, using normative data as outlined by Cameron and Dillon (2007).

### Assessment of Cognitive Abilities

#### Nonverbal Intelligence

Nonverbal intelligence (NVIQ) was estimated using the Test of Nonverbal Intelligence (Brown et al. 1982). In this test, participants are required to determine relationships among black-and-white abstract line drawings and choose a response from among six possible alternatives. Age-scaled quotient scores are calculated based on the number of correct responses.

#### Working and Serial Short-Term Memory

Two aspects of memory (working and serial short term) were assessed using the digit span task (Semel et al. 2003), where participants must repeat strings of live voice presented digits, either forward (serial short-term memory) or backward (working memory). The digit string length progressively increases, until the participant fails at two attempts at a particular string length. Scoring is based on the number of correctly repeated strings.

#### Sustained Attention (Auditory and Visual)

The Brain Train Integrated Visual and Auditory Continuous Performance test was used to assess the sustained attention (Sandford & Turner 2014). In this task, participants are presented with interleaved auditory and visual digits “1” and “2” and are required to click the mouse of a desktop computer for seen/heard “1” stimuli and to ignore any seen/heard “2” stimuli. The test assesses a number of different aspects of attention, but for the purposes of this study, only the full-scale attention quotient (converted to *z* scores and referred to as “attention”) was considered. The full-scale attention quotient combines the auditory and visual attention quotients and is scaled to have a normative mean of 100 and standard deviation of 15.

Children, who were unable to complete the attention task, or who returned an invalid result, were assigned a *z* score of −6 (the lowest score observed clinically). Eleven children (five AP− and six AP+) had scores in this range, suggesting serious difficulties with sustained attention that were specific to the clinical group. These data were consequently retained.

#### Wheldall Assessment of Reading Passages: Reading Fluency

Reading fluency, as assessed using the Wheldall Assessment of Reading Passages (WARP) (Madelaine & Wheldall 2002), was used as a proxy for academic abilities because preliminary research (Tomlin 2014) suggested it correlated well with more standard measures of academic ability (Pearson’s *r* ranging between 0.53 [numeracy] to 0.65 [reading comprehension]). In the WARP, children were required to read three text passages as quickly and accurately as possible in a minute. The numbers of correctly read words per minute for each passage were averaged to obtain a single measure of oral reading fluency. Final scores were normed for age differences.

### Procedure

All behavioral testing took place in the Department of Audiology and Speech Pathology of the University of Melbourne. Once consent to participate was received, parents (from both the clinical and TD groups) completed the comprehensive case history developed in-house to ensure the children met recruitment criteria. They then completed the same test battery (outlined above), with order of presentation being randomized across children. Because of the length of the test battery, it was completed over two separate sessions approximately 2 weeks apart, and each lasting between 1 and 1.5 hr.

Parents completed the FAPC and ECLiPS questionnaires, while the child was being assessed. The clinician and child completed the LIFE together at the time of assessment. Parents asked the child’s teacher to complete the TEAP between the two assessments. In some cases, parents chose not to involve their child’s teacher in the study. This resulted in some missing TEAP data.

### Data Analysis

Most statistical analyses were conducted in SPSS version 19 for Windows (2010). Descriptive statistics were calculated (Table 2) for each variable to test for deviations from normality (Kolmogorov–Smirnov tests *p* > 0.05, z-skewness and/or z-kurtosis >2.5 or <−2.5). Almost all variables deviated from normality, and Spearman’s rho, *r*_*s*_, is reported for all correlations. Most variables satisfied criteria for within-group assessments of normality (Kolmogorov–Smirnov tests *p* > 0.05), and parametric analyses were applied for between-group analyses. Group sizes are small for chi-square tests, and these analyses were performed in R (R Core Team 2014) applying Barnard’s Monte Carlo test (Barnard 1963).

**TABLE 2.**
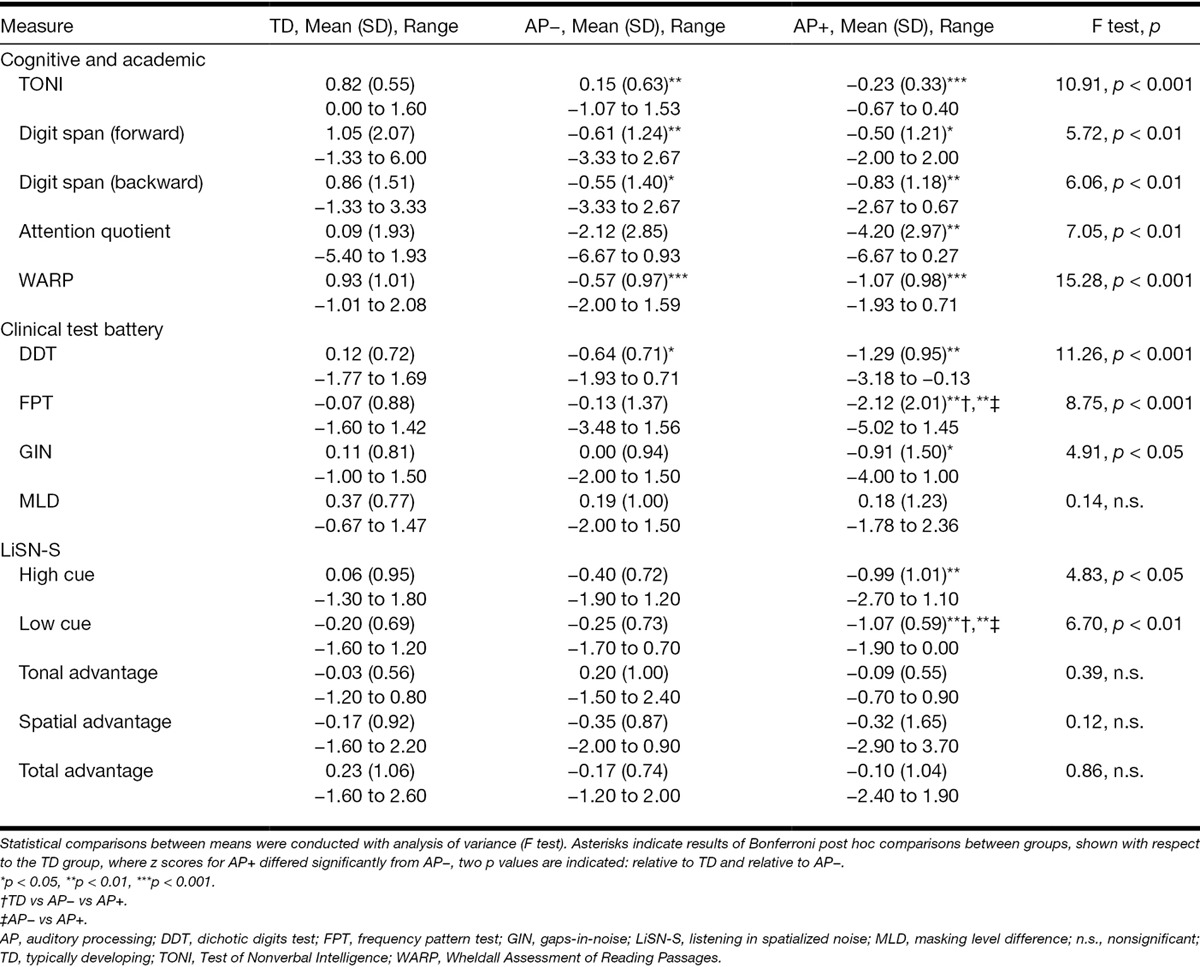
Performance on behavioral measures (mean z score, SD, and range) for the participants, subdivided according to recruitment location (TD/clinical) and within the clinical group, according to diagnosis (AP−/AP+)

## RESULTS

### Comparison of Group Performance on All Measures

Table 2 compares mean observations for all behavioral measures for the three subgroups of children in the study (TD, AP−, and AP+), while Table 3 compares the scores for the three subgroups on the questionnaires.

**TABLE 3.**
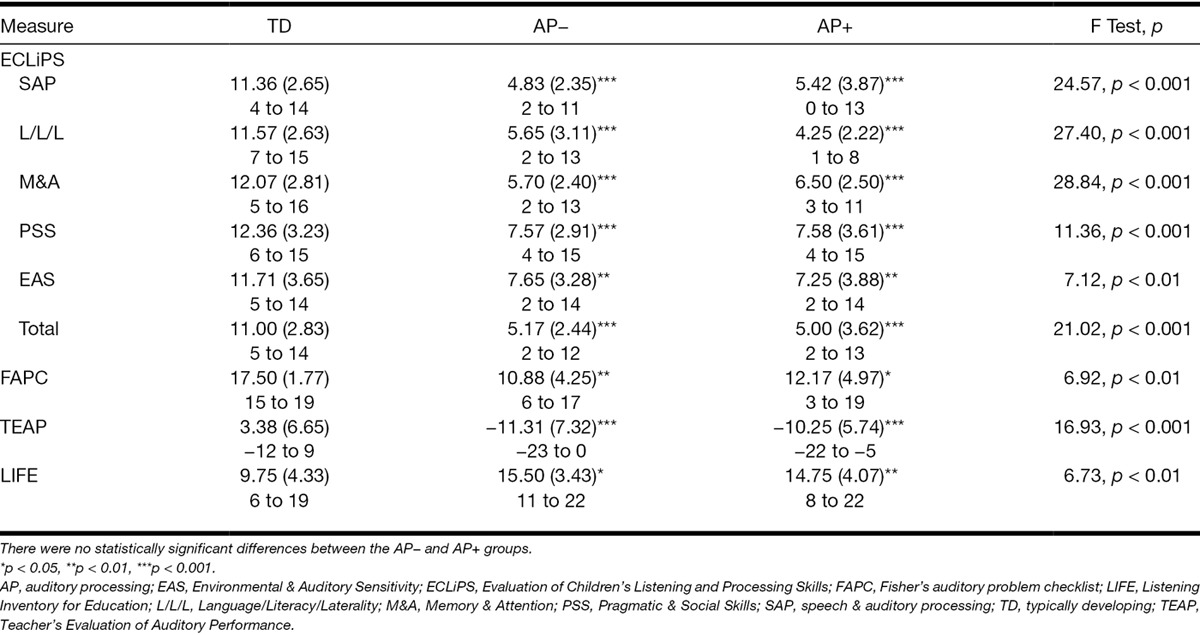
Comparison of questionnaire scores (mean, SD, and range) in the study for the three groups of children with respect to the TD group

**Fig. 1.**
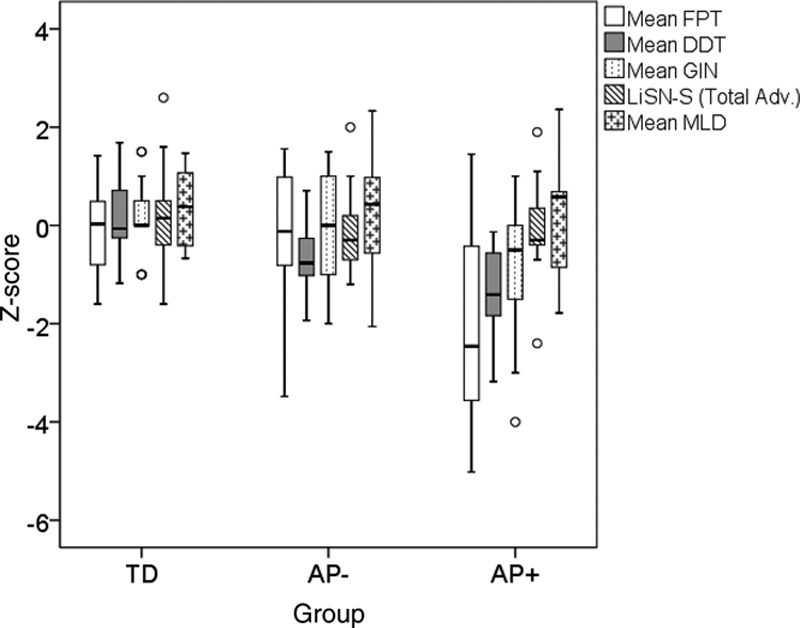
Box plot summary of performance on the different clinical auditory processing (AP) tests for the subgroups (typically developing [TD]; AP−; AP+). The boxes encompass the interquartile range of performance, with median performance indicated by the thick line. The whiskers show the range of performance, with outliers (o) defined as points more than 1.5 box lengths away from the upper or lower edge. DDT indicates dichotic digits test; FPT, frequency pattern test; GIN, gaps-in-noise; LiSN-S, listening in spatialized noise; MLD, masking level difference.

The TD group scored more highly than either of the clinical subgroups on all clinical AP measures except MLD and the LISN-S tonal, spatial, and total advantage scores. The AP− subgroup was not statistically different from the TD group on either the attention quotient measure or any of the clinical AP measures except the DDT. By contrast, the AP+ subgroup was distinct because of both poorer performance on the clinical test battery (by definition) and poorer mean scores for attention [*F*(2,43) = 7.05, *p* < 0.01, Bonferroni *post hoc* tests, AP+ < TD (*p* < 0.001), AP+ < AP− (*p* = 0.06)]. Figure 1 summarizes the performance on key measures from the clinical test battery.

The clinical subgroups were rated as having more difficulties than the TD group on all questionnaires (Table 3). Given their diagnostic status, one might predict that the AP+ would emerge as having greater listening difficulty than the AP− group. In fact, this prediction was not supported by the data. There was nothing in any of the questionnaires to suggest either quantitative or qualitative differences in symptoms between the two clinical subgroups. The groups were therefore treated as a single group (referred to as “clinical”) for all subsequent correlational analyses.

### Convergence Among Questionnaires

The five ECLiPS factors (SAP, EAS, L/L/L, M&A, and PSS) together with the ECLiPS total score were entered into correlational analyses with the three other questionnaires (LIFE [self], TEAP [teacher], and FAPC [parent]). Observations are summarized in Table 4. From these data, the following points can be made.

**TABLE 4.**
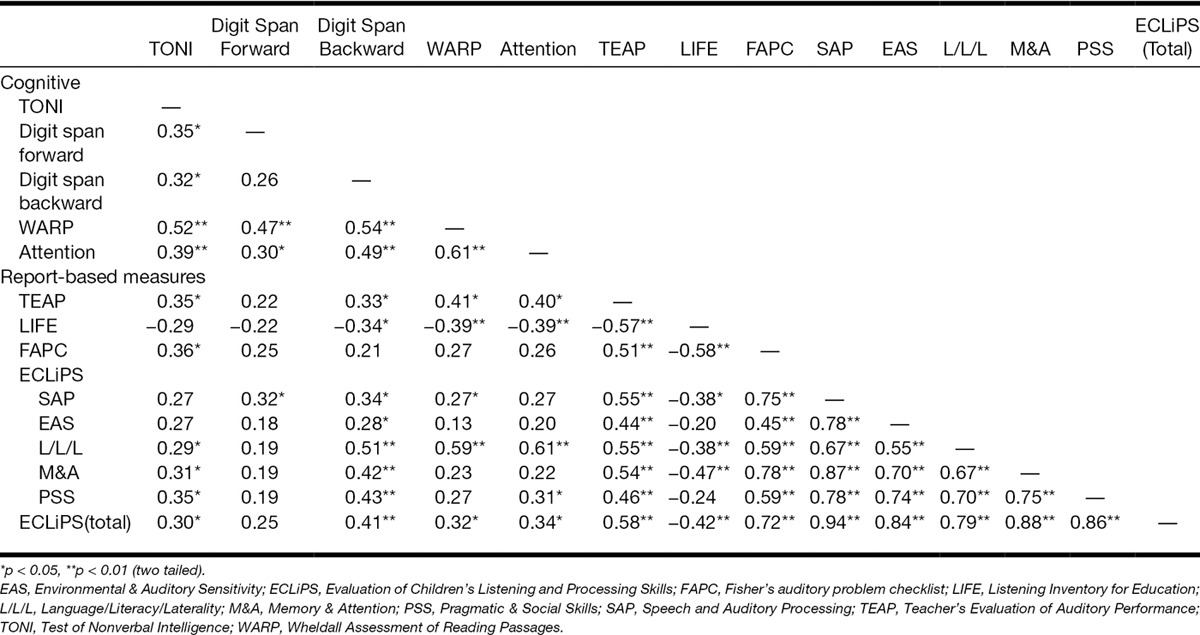
Summary of correlation analyses between the cognitive, academic, and questionnaire-based measures

First, the five ECLiPS factors correlate highly with each other and with the ECLiPS(total) score. Excluding the correlations with the ECLiPS(total) score, the highest correlation coefficient was between SAP and M&A (*r*_*s*_ = 0.87) and the lowest was between L/L/L and EAS (*r*_*s*_ = 0.55). It is not surprising that the five factors correlate, given that an oblique rotation was applied during their extraction (Barry & Moore 2014). However, the high correlation coefficient between SAP and M&A is noteworthy because it suggests either that the two factors are measuring the same latent trait or that they are probing two different but closely related traits.

Second, significant correlations were observed between the three other questionnaires. Coefficients ranged from *r*_*s*_ = 0.51 (FAPC versus TEAP) to *r*_*s*_ = −0.58 (FAPC versus LIFE).

**Fig. 2.**
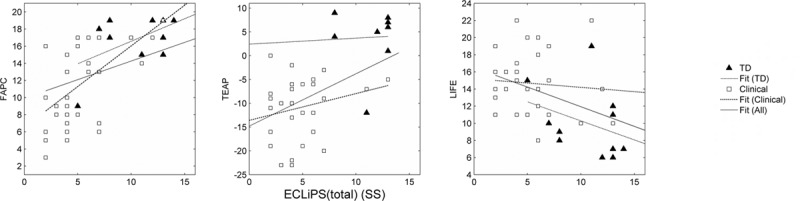
Correlations between the Evaluation of Children’s Listening and Processing Skills (ECLiPS(total)) (standard score [SS] *x* axes)) and Listening Inventory for Education (LIFE), Teacher’s Evaluation of Auditory Performance (TEAP), and Fisher’s auditory problem checklist (FAPC) (raw scores, *y* axes) for the typically developing (TD) (▴) and clinical groups (▫). Fit lines are provided for each group separately (dashed/dotted lines) as well as for all participants (solid line).

Third, with the exception of EAS and PSS versus LIFE (*r*_*s*_ = −0.20), all ECLiPS measures correlated significantly with the LIFE, TEAP, and FAPC (Table 4). Scatter plots of the ECLiPS(total) score versus the TEAP, FAPC, and LIFE are provided in Figure 2, with fit lines for the data as a whole and for the groups subdivided according to recruitment. It is apparent from these plots that the correlations emerging between TEAP/LIFE scores and the ECLiPS(total) scores for the combined groups are primarily a consequence of differences between the groups. There was little evidence for within-group relationships for these variables.

Greatest convergence (*r*_*s*_ > 0.7) is apparent between the FAPC and three ECLiPS scores (total, SAP, and M&A factors; Table 4). This convergence probably reflects the fact that the FAPC and ECLiPS are both parental-report measures, and the FAPC focuses on difficulties similar to those captured by the SAP and M&A factors of the ECLiPS.

### Convergence Between Clinical AP Tests and Questionnaires

Previous research (Lam & Sanchez 2007; Wilson et al. 2011) suggested that few, if any, correlations would be observed between performance on the tests typically included in clinical test batteries, and questionnaires such as the FAPC, TEAP, or LIFE. These questionnaires incorporate a range of different presenting symptoms within a single mean score. This mean score is therefore likely to reflect contributions from a range of latent abilities, not all of which will be relevant for explaining the performance on clinical AP tests.

Unlike the other questionnaires, the ECLiPS, with its five-factor structure, provides scores for separate, albeit related, latent traits. Convergence with AP measures would therefore potentially emerge with scores for factors relying on similar latent traits (abilities). We specifically predicted that the L/L/L and M&A factors would associate with clinical AP measures where task performance also reflected support from these cognitive abilities.

Replicating, Lam and Sanchez (2007) and Wilson et al. (2011), no significant correlations were observed between any of the test battery measures and either the LIFE, TEAP, or FAPC (*r*_*s*_ all < 0.28) (Table 5). Correlations, however, reached significance between the mean DDT score and three ECLiPS factors: SAP (*r*_*s*_ = 0.29, *p* < 0.05), L/L/L (*r*_*s*_ = 0.31, *p* < 0.05), and M&A (*r*_*s*_ = 0.30, *p* < 0.05).

**TABLE 5.**
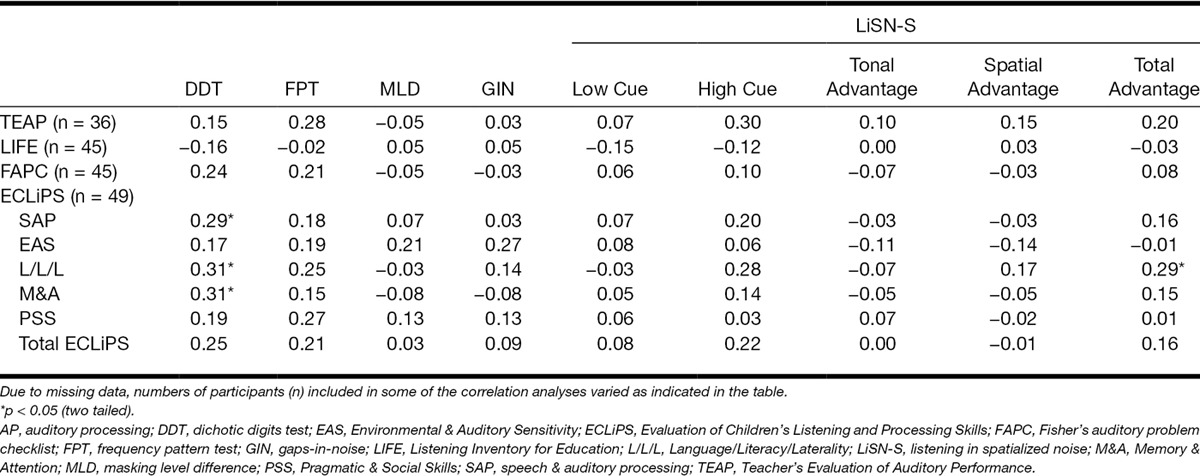
Summary of correlation analyses between the clinical AP test battery and the questionnaires in the study

**Fig. 3.**
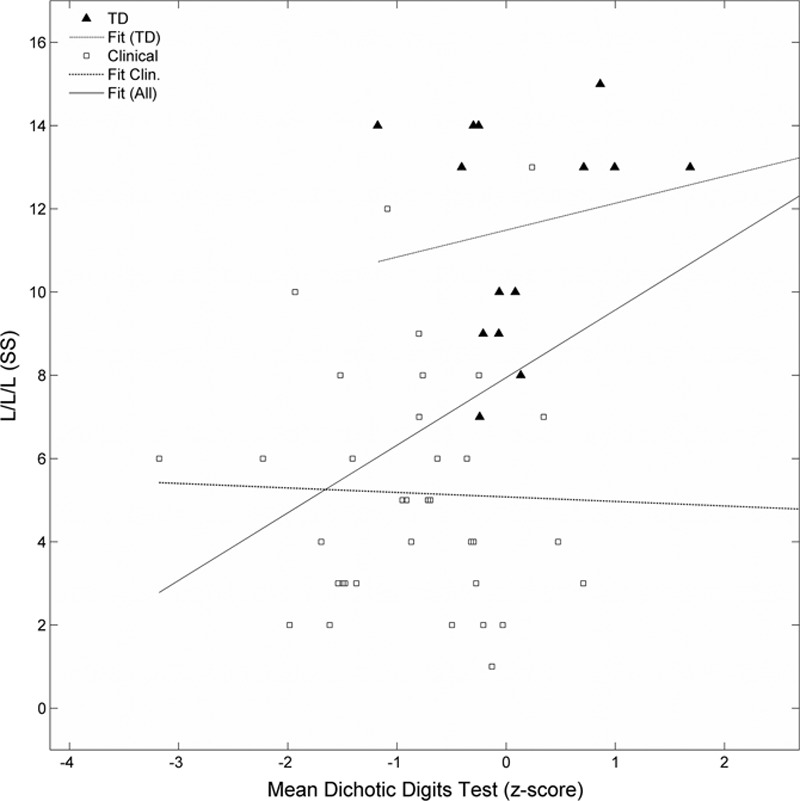
Plot of standard scores (SS) for the Evaluation of Children’s Listening and Processing Skills Language/Literacy/Laterality (L/L/L) factor against *z* scores for mean performance on the dichotic digits test. TD indicates typically developing.

Scatter plots of the data (e.g., L/L/L versus DDT; Fig. 3) did not suggest that the ECLiPS had any within-group power to predict the performance on the DDT for either the clinical or the TD groups. This was confirmed with a multiple regression analysis showing that while group membership significantly predicted the L/L/L score (*p* < 0.001), the mean dichotic digit score did not (*p* = 0.88). The significant correlations observed in the combined data are thus attributable to the largely nonoverlapping distributions of data for the two groups of participants.

### Convergence/Divergence of Questionnaires With Reading Fluency/Cognitive Abilities

Academic and/or underlying cognitive difficulties manifesting as listening difficulties may contribute to a child being referred for clinical assessment. In this next series of analyses, the relationship between these difficulties and the questionnaires was assessed.

#### Associations Between Reading Ability (WARP) and Questionnaires

The TEAP (teacher) and LIFE (self) assess listening in the school context, and significant correlations were observed between the WARP and scores on both these measures (TEAP, *r*_*s*_ = 0.41, *p* < 0.05; LIFE, *r*_*s*_ = −0.39, *p* < 0.01). Scatter plots suggest some relationship between the WARP and these questionnaires in the TD group, but not in the clinical group. Significance thus largely emerges because of differences between the groups.

**Fig. 4.**
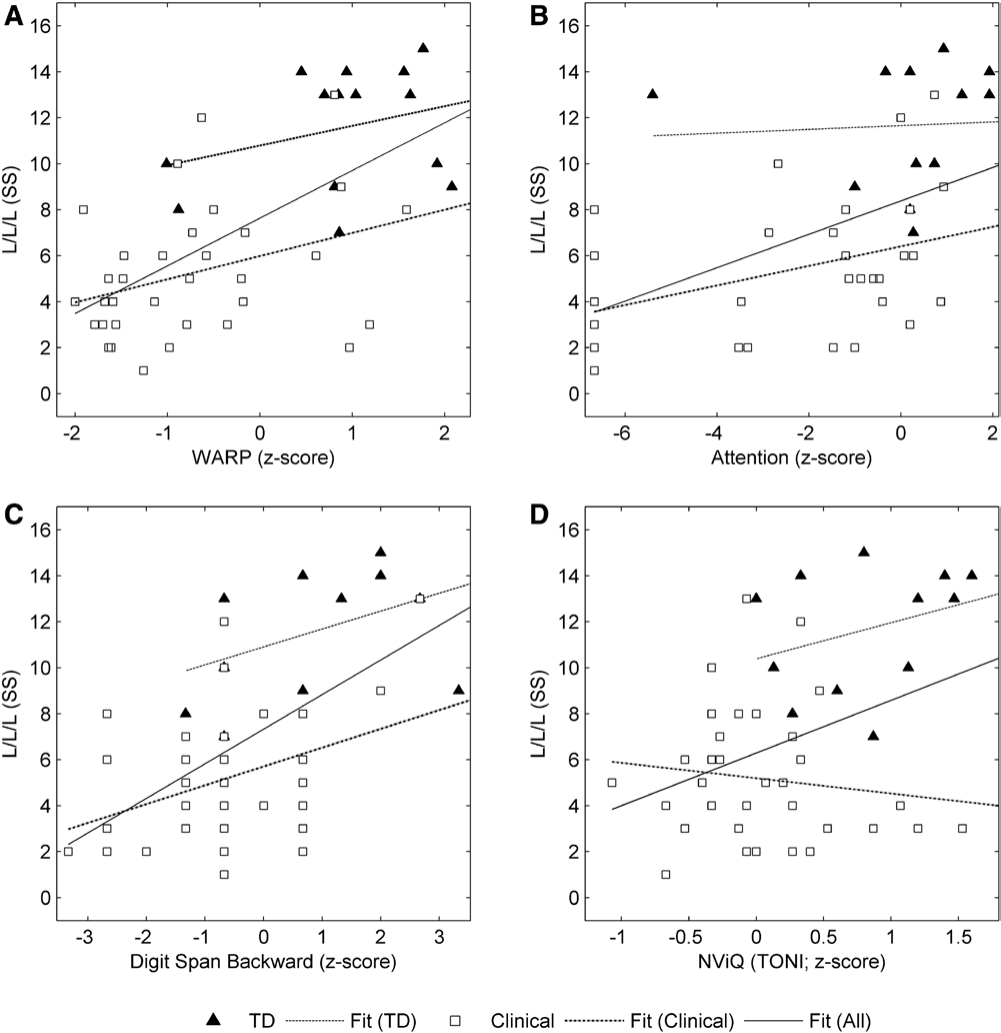
Plot of Evaluation of Children’s Listening and Processing Skills (Language/Literacy/Laterality [L/L/L]) (standard score [SS]) against *z* scores for the Wheldall Assessment of Reading Passages (WARP) (A), attention (B), digit span backward (C), and nonverbal intelligence (NVIQ) (D). TD indicates typically developing.

No significant relationship was observed between the FAPC (parent report) and the WARP (*r*_*s*_ = 0.27). This relative lack of significant relationship suggests that the FAPC score may capture difficulties that are not necessarily specific to academic performance. This contrasts with the ECLiPS (also a parental-report measure) where significant correlations were observed between the WARP and the factors: L/L/L (*r*_*s*_ = 0.59, *p* < 0.001) and SAP (*r*_*s*_ = 0.30, *p* < 0.05) as well as the ECLiPS(total) score. Scatter plots (e.g., L/L/L versus WARP; Fig. 4A) suggest that significance largely depends on the combination of two groups with different distributions of data, though a trend is apparent (*p* = 0.06) between the WARP scores and the L/L/L factor in the clinical group.

#### Relationships Between Questionnaire-Based Measures and Cognitive Abilities

Correlations (Table 4) were performed between responses from the questionnaires and the four measures of cognitive abilities.

Digit span backward correlated significantly with TEAP, LIFE, and all ECLiPS scores. By contrast, digit span forward correlated significantly only with the ECLiPS SAP factor (*r*_*s*_ = 0.32, *p* < 0.05). Attention correlated most highly with the L/L/L factor (ECLiPS). Test of nonverbal intelligence-4 correlated with the TEAP and FAPC and all ECLiPS factors, except SAP and EAS.

Scatter plots of L/L/L versus attention (Fig. 4B), digit span backward (Fig. 4C), and, to some extent, NVIQ (Fig. 4D) suggested that correlations not only reflected between-group differences but also some within-group effects. This was confirmed by multiple regression, which showed that the L/L/L score remained significantly correlated with attention and digit span backward (*p* = 0.03) even when group membership was included in the regression.

### Which Measures Optimally Discriminate Among Participant Groups in the Study?

A key aim for this work was to consider what role, if any, the ECLiPS could play in supporting the clinical assessment of children referred because of suspected APD.

All the questionnaires in the study were similar in indicating some form of difficulty (i.e., scores for the clinical groups were significantly lower than for the TD group). The preceding analyses did, however, suggest that the responses on the ECLiPS were more likely than the other questionnaires to relate to performance on the clinical AP and cognitive measures in this study. This provides some evidence for construct validity (i.e., it captures issues relevant to both types of measure), but it does not address the question of whether the ECLiPS contributes more or less useful information than the other questionnaires to the clinical assessment process. To address this latter question, “useful information” was operationalized “as most efficient classification of participants into their respective groups (TD, AP−, and AP+).” Thus operationalized, the question was assessed through a series of discriminant analyses.

Discriminant analysis aims at determining the best linear combination of variables required for predicting the group membership of a set of individuals. For all analyses performed here, variables were entered using a stepwise procedure and Wilks’ lambda (F-ratio, 3.84–2.71) was applied as the decision criterion for determining each variable’s entry and removal from the procedure. Prior probabilities were calculated from the original sizes of the groups entered, and missing data were replaced by mean data.

#### Analysis 1: Group Classification Using the Clinical AP Test Battery

The first analysis considered which linear combination of clinical AP tests contributed significantly to group classification. Two tests were identified, namely, DDT and FPT. These combined to form two orthogonally arranged discriminant functions, with most variance (canonical *R*^2^ = 0.508) accounted for by the first function. Combined, the two functions successfully classified 59.3% of the children into their original groups. Most classification errors were between the TD and AP− groups (Table 6) and reflected the overlap in AP abilities of the children in these two groups. A minority of children (3) in the AP+ group were also not correctly classified because their status as AP+ reflected poor performance on AP tests other than DDT and FPT.

**TABLE 6.**
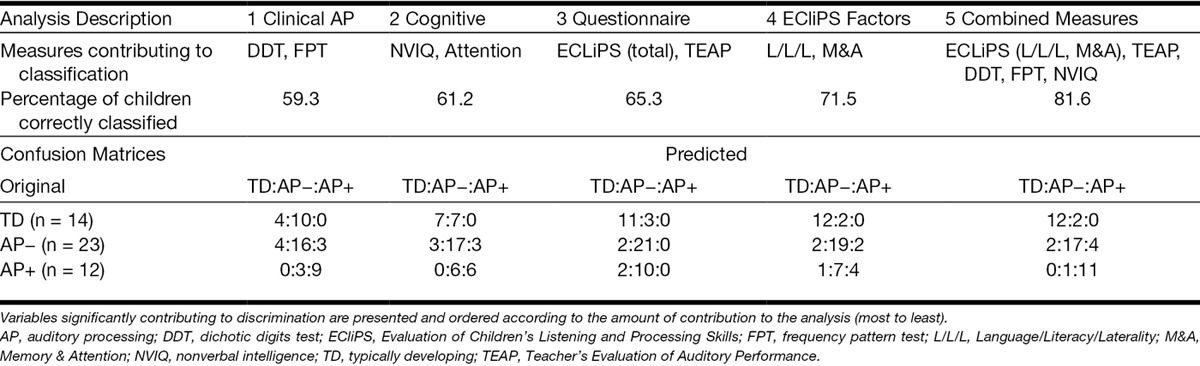
Summary of the discriminant analyses performed

#### Analysis 2: Group Classification Using Cognitive Measures Only

Only two of the four cognitive measures (NVIQ and attention) significantly contributed to discriminant analysis 2. Relative to analysis 1, there was a slight improvement in overall classification accuracy (61.2%). The improvement reflected fewer errors in the classification of the TD and AP− groups (Table 6) but a greater number of errors distinguishing between AP+ and AP− children.

#### Analysis 3: Group Classification Using Questionnaires (FAPC, ECLiPS(Total), TEAP, and LIFE)

Two questionnaires (ECLiPS(total) and TEAP) significantly contributed to discriminant analysis 3. Overall, 65.3% of the children were classified into their original groups. As one might expect given that the clinical groups did not differ in presenting symptoms (Table 3), neither of the two canonical functions extracted as part of this analysis could discriminate between the AP+ and AP− groups (Table 6). Group classification depended entirely on the first canonical function which was more efficient than the AP measures (analysis 1) in separating the TD children from the clinical children.

#### Analysis 4: Group Classification Using the Five ECLiPS Factors

Two ECLiPS factors (L/L/L and M&A) significantly contributed to analysis 4. The two canonical functions that they formed successfully classified the three groups of children into their original groups with 71.5% accuracy. Unlike the questionnaires in analysis 3, the combination of variables in analysis 4 was able to discriminate to a limited extent between the AP+ and AP− groups (Table 6). Examination of the loadings of L/L/L and M&A on to the second canonical function suggested that this increased power to discriminate between the groups reflected a tendency for the AP+ group to have lower scores on the L/L/L factor relative to the M&A factor.

#### Analysis 5: Group Classification Using a Combination of Measures

The final discriminant analysis involved inclusion of only those AP (DDT and FPT), cognitive (NVIQ and attention), and report-based measures (ECLiPS [L/L/L and M&A] and TEAP) that the preceding analyses (1–4) suggested contributed to classification. Attention ceased to contribute once the questionnaires were added to the analysis, suggesting that the variance associated with attention was effectively accounted for by them.

**Fig. 5.**
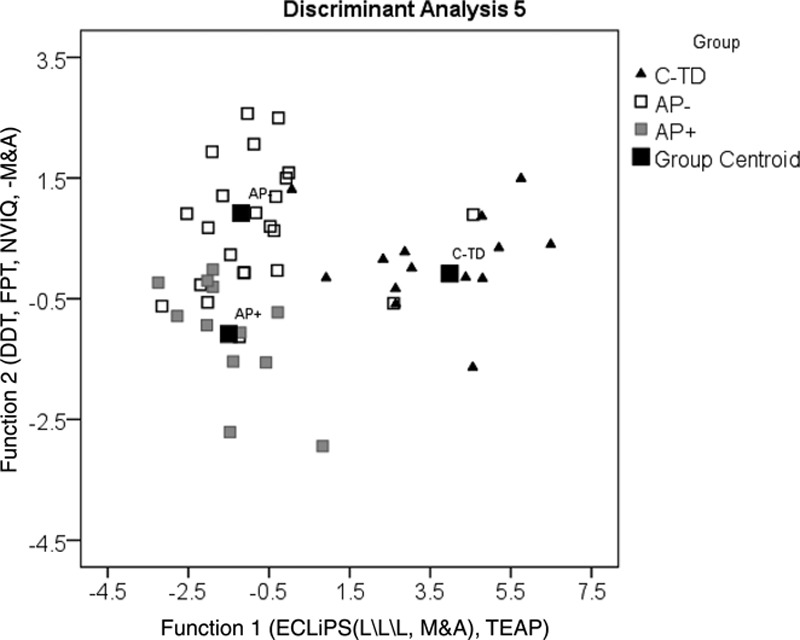
Final discriminant analysis showing how the two extracted functions separated the groups. Function 1, predominantly reflecting contributions from Evaluation of Children’s Listening and Processing Skills (ECLiPS) (Language/Literacy/Laterality [L/L/L] and Memory & Attention [M&A]) and Teacher’s Evaluation of Auditory Performance (TEAP), separated the typically developing (TD) from the clinical groups. Function 2, predominantly reflecting the contributions from dichotic digits test (DDT), frequency pattern test (FPT), nonverbal intelligence (NVIQ), and M&A, separated the AP− from the AP+ and TD groups. AP indicates auditory processing.

The canonical functions based on the remaining six variables both significantly contributed to correct classification of 81.6% of the children into their original groups. The first function largely reflected the influence of the questionnaires, while the second reflected inputs from the cognitive and AP measures (Fig. 5).

## DISCUSSION

The primary goal of this study was to determine whether the ECLiPS (a recently developed questionnaire [Barry & Moore 2014]) contributed anything more to clinical assessment than three other currently available questionnaires (FAPC, TEAP, and LIFE).

Ideally, any instrument used for clinical diagnosis should be highly sensitive to, and specific for, the disorder of interest, as well as meeting psychometric requirements of measurement reliability (i.e., high test–retest reliability). Such a tool would represent a form of measurement gold standard. No such gold standard currently exists for diagnosing APD though it has been suggested that a psychometrically robust report-based measure of difficulty could potentially provide such a standard (Moore et al. 2013). This hope provided the primary motivation for the development of the ECLiPS. However, when the four questionnaires in this study were compared directly with each other, all seemed to be similarly sensitive to the presence or absence of some form of difficulty requiring referral. None, though, could discriminate between children meeting diagnostic criteria for APD and those not.

The fact that scores on all the questionnaires were lower for the children referred for clinical assessment relative to the TD children was to be expected. Of more interest was the lack of any apparent difference in severity of symptoms between the children satisfying the diagnostic criteria for APD (AP+) and those not (AP−). This finding further underlines one of the central challenges for clinicians assessing children: the lack of a consistent relationship between children with qualitatively similar symptoms and their subsequent performance on clinical AP test batteries (Moore et al. 2010). There are many possible reasons for this weak association between qualitative and objective measures of listening ability. First, with respect to the qualitative measures (questionnaires), in addition to whether or not they include the relevant range of items, estimates of severity are inherently imprecise and responses will be influenced by many factors, including a respondent’s personal interpretation of an item’s meaning, their ability to reliably gauge severity, and their possible desire for some sort of diagnosis. Second, the clinical AP tests may either lack construct validity (i.e., not measure the latent trait(s) relevant for APD) or may have construct validity, but incorporate noise due to contributions from non–auditory-specific factors such as cognitive ability or environmental experience (c.f., Bishop et al. 1999; Barry et al. 2012; Barry et al. 2013) or attention (Moore et al. 2010). It is notable, in this context, that the children meeting diagnostic criteria for APD were characterized by both lower (almost statistically significant) NVIQ and poorer attention than the AP− group. Third, qualitatively similar symptoms of difficulty could potentially develop out of many different underlying causes, and clinical AP tests may detect only the subset of children whose presenting symptoms specifically derive from AP deficits.

All of this invites the question: what is APD? The name suggests a single disorder, and some definitions have been very explicit about the range of difficulties included within this disorder (ASHA 2005; AAA 2010). While conceptually clear, numerous studies underline the difficulty of identifying children whose deficits are restricted to the auditory system (e.g., Sharma et al. 2009; Moore et al. 2010; Miller & Wagstaff 2011). For these reasons, Dillon et al. (2012) argue that rather than defining a single disorder, the term APD should be viewed as an umbrella term for a range of different AP deficits that ultimately affect ability to understand spoken language.

Unlike the other questionnaires used in this study, the ECLiPS, in addition to providing a single score for difficulty, also provides scores for five domain-specific clusters of symptoms (factors) that may be observed in children referred for suspected APD. Correlation analyses with clinical AP tests and these five domains provided evidence for a relationship between the DDT and three factors on the ECLiPS that tap into listening, language, and attention/memory skills. The DDT involves speech stimuli and probes the capacity to selectively attend to specific stimuli. The fact that this test correlates with factors on the ECLiPS tapping into traits involved in this task (listening [SAP], language [L/L/L], and memory and attention [M&A]) provides some evidence of construct validity for the factors.

In contrast with the clinical AP tests, a number of cognitive tests showed convergence with the questionnaires in the study. This suggests that cognitive difficulties may contribute to the pattern of presenting symptoms that lead to referral for clinical assessment (see also Tomlin et al. in revision). Cognitive difficulties, however, are also associated with poorer AP scores and hence with an increased likelihood of receiving a diagnosis of APD. We cannot currently tell whether these are simply associations arising from a common cause or whether the cognitive deficits directly contribute to poor scores on the AP tests. Though, in support of this last interpretation, it is notable that group differences were not observed for the derived measures, that is, MLD and LiSN-S (spatial, tonal, and total advantage scores). These scores are based on the subtraction of two measures that are assumed to place the same cognitive demands on the child (e.g., Moore et al. 2010).

An interesting pattern of correlations was observed between some of the ECLiPS factors and the cognitive (digit span backward and sustained attention) and academic (WARP) measures. Specifically, all ECLiPS factors, but most notably the language-specific factor (L/L/L), correlated with digit span backward. This test probes working memory, which has been shown to play a crucial role in both language learning and academic success in young children (Gathercole et al. 2005). It is notable in this context that the correlation between L/L/L and WARP approached significance in the clinical group alone and was not wholly a reflection of two different distributions of data for the clinical and TD groups.

With respect to sustained attention, again the strongest correlation was observed with the ECLiPS language factor (L/L/L). This is also consistent with a range of evidence suggesting a link between sustained attention and the development of language skills (Spaulding et al. 2008; Ebert & Kohnert 2011; Dispaldro et al. 2013).

In summary, significant correlations between the ECLiPS and the other three questionnaires suggested that the measures largely captured similar information about range and severity of presenting symptoms related to listening difficulty. However, unlike the TEAP, LIFE, and FAPC, the ECLiPS was able to provide more information about which clusters of presenting symptoms associated with individual cognitive difficulties. This suggests that the ECLiPS has the potential to support clinical practice by providing a rapid preliminary screen for cognitive difficulties, which may be contributing factors in both listening and language difficulties.

### Optimal Assessment: A Combination of AP, Cognitive, and Report-Based Measures

A series of discriminant analyses were performed to assess how well each kind of measure separately, as well as in combination, was able to correctly classify the three groups of children. No single group of measures (AP, cognitive, or questionnaire) was particularly good at classifying the children though the questionnaires alone (specifically the ECLiPS, L/L/L, and M&A factors) performed a little better than either the AP or cognitive measures alone. This relative success reflected a better separation of the TD children from the clinical group, as well as some successful classification of individuals from the clinical group into the AP+ and AP− subgroups.

The AP measures alone were unable to correctly classify many children in the TD and AP− groups. This underlines both the wide variation in AP test performance in these two groups, as well as the weak relationship between symptoms of listening difficulty leading to referral for an APD assessment and subsequent performance on the AP tests making up that assessment test battery.

Overall, the discriminant analyses undertaken in this study suggested that an optimal test battery for APD would comprise a combination of cognitive, auditory, and report-based measures, with the report-based measure of choice for achieving the best classification of the children, being the ECLiPS (specifically the L/L/L and M&A factors).

It is important to note, however, that the analyses presented here were based on a relatively small sample. Though the results support the use of the ECLiPS in combination with cognitive and AP measures, they need to be confirmed with a larger group of children whose diagnostic status is not predetermined as part of the analysis.

### Further Validation of the ECLiPS

Questionnaires are commonly included in APD assessment protocols (Emanuel et al. 2011), yet few, if any, have been rigorously assessed for psychometric reliability or validity. This apparent lack of interest in such issues among clinicians has likely had a negative impact on the reputation of questionnaires as reliable, informative measurement tools.

In the case of the ECLiPS, achievement of psychometric validity, including construct validity, was a central aim in the development of the questionnaire. The present study, with its focus on cognitive and clinical AP measures, represented a second stage in the assessment of construct validity. Correlations were observed between the ECLiPS factors and some AP measures, which were not also seen with the TEAP, LIFE, and FAPC. The work is based on relatively small numbers of participants, which reduces the power of the study to show the relationships between measures. Nonetheless, the findings with respect to the TEAP, LIFE, and FAPC are consistent with those from other studies involving similar measures but larger numbers of participants (e.g., Tomlin et al. in revision; Wilson et al. 2011). This gives us confidence that the correlations observed with the ECLiPS have some validity. In future research, it will be interesting to further verify the findings reported here with larger numbers of children.

This study has considered construct validity in the ECLiPS relative to cognitive and AP measures. However, validation is a continuous process of comparison with many different types of measure tapping into related and unrelated traits (Campbell & Fiske 1959). Such a process is particularly important in the context of APD, where we lack a gold standard benchmark. Physiological (e.g., Sanches & Carvallo 2006) and electrophysiological measures (e.g., Muchnik et al. 2004; Sharma et al. 2014) have also been identified as relevant to APD. In addition to behavioral studies based on larger number of children, assessment of construct validity relative to these other objective measures of listening difficulty will represent an important next step in the questionnaire’s development.

### Conclusion: The Role of the ECLiPS in Clinical Assessment

There is currently considerable dissatisfaction with the process of assessing and diagnosing children referred for suspected APD. Part of the problem reflects the lack of a gold standard test for the disorder resulting in a debate about which AP tests should be included in a clinical test battery, how many tests should be included, and what cutoff criteria should be applied for establishing a diagnosis. Dillon et al. (2012) have argued for a systematic and hierarchical approach to assessment to address some of these issues. The first stage of Dillon et al.’s (2012) proposed assessment protocol aims at first determining whether a child requires further assessment by a hearing specialist. Questionnaires can play an important role in this decision-making process. All the questionnaires in this study were all similarly sensitive to the presence of a listening difficulty. However, the ECLiPS, by virtue of its five-factor structure, offered relatively more information about potential underlying cognitive or language difficulties that might manifest as listening difficulty. Such information is useful for deciding whether referral to a hearing specialist alone is appropriate, and if not, which other specialists should be involved in the assessment and subsequent management of the referred child.

From a research perspective, discriminant analysis 4 (Table 6) suggests that information captured within individual factors in the ECLiPS can be exploited to further understand what differentiates children who meet commonly applied criteria for a diagnosis of APD, from those who do not.

Finally, unlike other commonly used questionnaires, the ECLiPS is distinct in having well-understood psychometric properties (Barry & Moore 2014) and being easily understandable by respondents with a broad range of reading abilities—a recognized failing of a number of questionnaires in this field (Atcherson et al. 2013).

This study has focused on issues regarding the construct validity of the ECLiPS—a new questionnaire—and its possible role in supporting the assessment of children referred for APD. The results are encouraging, but more research is needed involving larger-scale studies applied in clinical contexts where the benefits of the questionnaire in supporting clinical decisions regarding the appropriateness of referral, or regarding the design of a management plan subsequent to assessment, can be more extensively explored.

## ACKNOWLEDGMENTS

The Evaluation of Children’s Listening and Processing Skills (ECLiPS) questionnaire was developed by Johanna G. Barry and David Moore. It is commercially available in the United Kingdom.
